# Palaeolithic voyage for invisible islands beyond the horizon

**DOI:** 10.1038/s41598-020-76831-7

**Published:** 2020-12-03

**Authors:** Yousuke Kaifu, Tien-Hsia Kuo, Yoshimi Kubota, Sen Jan

**Affiliations:** 1 The University Museum, The University of Tokyo, Tokyo, 113-0033 Japan; 2 Institute of Oceanography, National Taiwan University, Taipei, 10617 Taiwan; 3 Department of Geology, National Museum of Nature and Science, Ibaraki, 305-0005 Japan

**Keywords:** Archaeology, Physical oceanography

## Abstract

How Palaeolithic maritime transportation originated and developed is one of the key questions to understand the world-wide dispersal of modern humans that began 70,000–50,000 years ago. However, although the earliest evidence of maritime migration to Sahul (Australia and New Guinea) has been intensively studied, succeeding development of Paleolithic maritime activity is poorly understood. Here, we show evidence of deliberate crossing of challenging ocean that occurred 35,000–30,000 years ago in another region of the western Pacific, the Ryukyu Islands of southwestern Japan. Our analysis of satellite-tracked buoys drifting in the actual ocean demonstrated that accidental drift does not explain maritime migration to this 1200 km-long chain of islands, where the local ocean flows have kept the same since the late Pleistocene. Migration to the Ryukyus is difficult because it requires navigation across one of the world’s strongest current, the Kuroshio, toward an island that lay invisible beyond the horizon. This suggests that the Palaeolithic island colonization occurred in a wide area of the western Pacific was a result of human’s active and continued exploration, backed up by technological advancement.

## Introduction

The rise of voyaging technology beyond nearshore boating was a key for early modern humans to exponentially expand their habitable territory on the globe. Maritime migration to Sahul (a combined continent of Australia and New Guinea), which occurred about 47,000 years ago or earlier, is the oldest accepted evidence for open ocean crossings by modern humans^1–5^, and has been central to such discussion. There is growing consensus that the colonization of Sahul was a consequence of deliberate voyages, based on theoretical considerations and circumstantial evidence such as the need of repeated sea-crossings, a more or less large number of immigrants needed to establish a viable population, and archaeologically demonstrated advanced maritime adaptation including pelagic fishing^4,6–9^ (but see ref.^10^ for a contrary view). Although the exact seaway taken by these Palaeolithic voyagers still remains undetermined, at least one main route ensured visibility of target islands all through the course to Sahul^11–14^. The densely distributed large islands and the warm sea surface temperature of Wallacea (eastern Indonesia) were advantageous for these earliest voyages.

**Figure 1:**
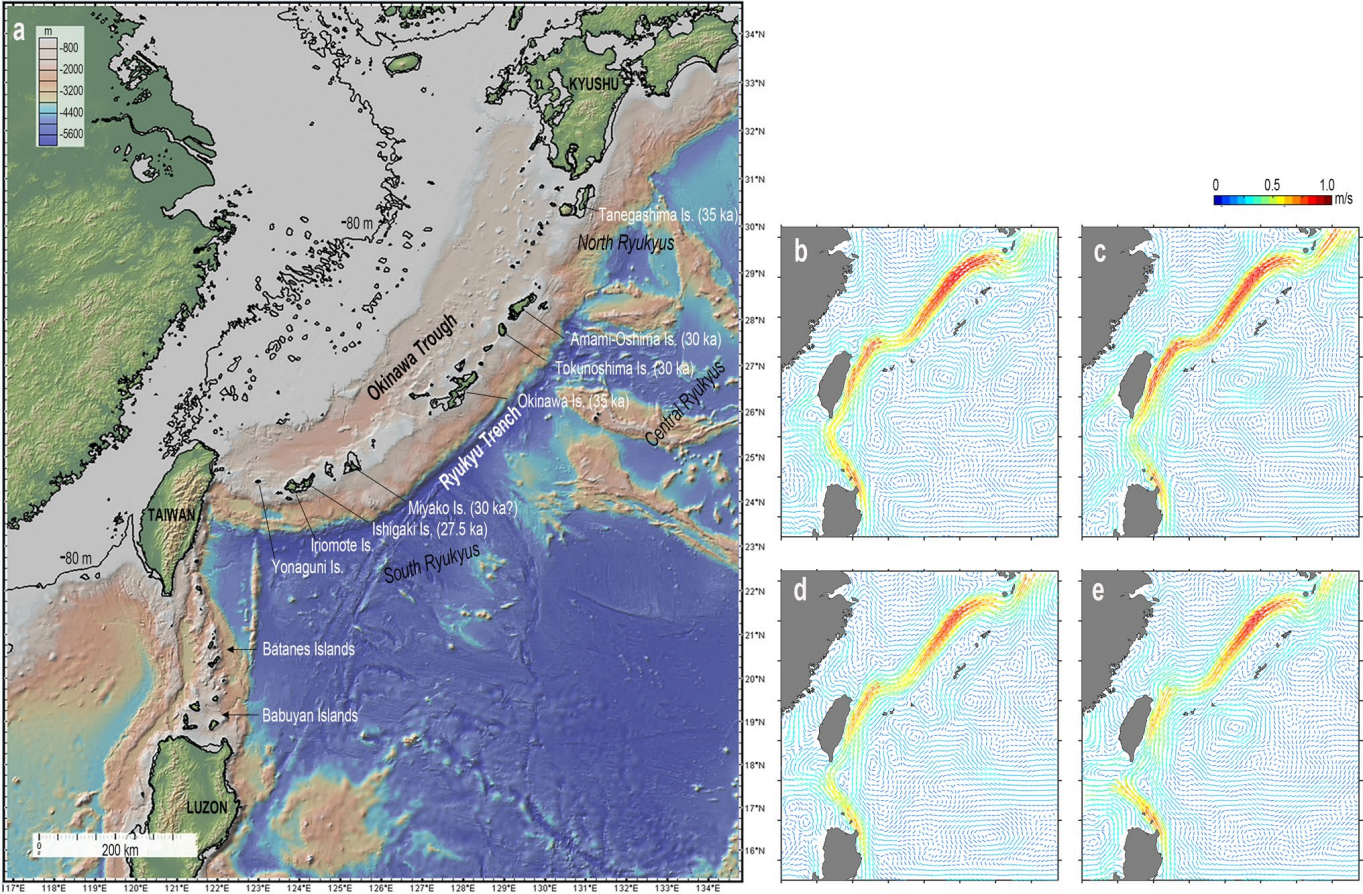
(**a**) Topography of the study area with the estimated coastlines 35,000–32,000 years ago. The oldest known calibrated ages of archaeological sites on six islands of the Ryukyus are within the parentheses (figure created using the GeoMapApp 3.6.10 software: https://www.geomapapp.org). (**b**–**e**) Seasonal mean circulation patterns in the study area calculated using 10-year (2009–2018) satellite altimetry sea surface heights derived absolute geostrophic currents obtained from the Archiving, Validation and Interpretation of Satellite Oceanographic data (AVISO) (figures created using the Matlab R2019a: https://www.mathworks.com/products/matlab.html?s_tid=hp_products_matlab). (**b**) Spring (March–May). (**c**) Summer (June–August). (**d**) Fall (September–November). (**e**) Winter (December–February).

However, the western Pacific holds other areas with evidence of sea crossings during the Marine Isotope Stage 3, which are equally important to understand the developmental processes of early maritime technology and activity. The Ryukyu Island Arc in southwestern Japan (Fig. 1) is particularly interesting in this context. Here, archaeological sites found on six different islands suggest that maritime migration occurred ~ 35,000–30,000 years ago both from north (via Kyushu) and south (via Taiwan)^15^. Migration to these islands is challenging. The islands are small, of low elevation and not all are intervisible. Moreover, one of the world’s largest and strongest ocean currents, the Kuroshio, intervenes the water way (Fig. 1).

At the southern entrance to the Ryukyu Islands, the Kuroshio is flowing poleward. The Kuroshio transports coconuts and various other floating objects to the islands of Japan from south. This observation inspired some earlier researchers to suggest that the first islanders of the Ryukyus (and possibly the Japanese main islands) arrived from south by accidental drifts with the Kuroshio^16^. In order to test the validity of this drift hypothesis, in this study, we examine the chance for a primitive watercraft drifted from the upstream of the Kuroshio (off the east coast of Taiwan or northern Luzon) to reach islands of the Ryukyu Arc.

One way to approach this question may be a computer-assisted simulation of drifting^17,18^. A recent such study used a 0.1° × 0.1° grid model for Wallacea and Sahul, and examined if drifting objects driven by forces from modeled modern ocean surface flows and winds successfully reach islands in the region^19^. The computed low success rate (< 5%) did not support the accidental drift hypothesis. However, although we agree that such simulations are useful, it is unclear how accurately they replicate the actual drifts at sea. For example, the accuracy of the ocean model used is often ambiguous; the grid size may be too large to reflect complicated and hourly changing ocean flows; other factors influencing a floating object such as wind waves and swells are not considered.

For these reasons, in the present study, we examine the trajectories of satellite-tracked surface drifting buoys deployed in the Global Drifter Program (formerly known as Surface Velocity Program (SVP): see “Methods” section). The advantage of using the SVP drifters are the large number of standardized, quality-controlled trajectories recorded in the actual ocean in all seasons and various sea surface conditions over the past 30 years or so. The forces (current, wind-driven surface flow, waves, and wind) which exert to an ancient watercraft vary depending on its size, shape, draft, as well as people and goods on board, and these are somewhat different from those drive SVP drifters. However, we expect such differences are small. At least, lesser wind effects on these drifters will be advantageous for them to reach islands of the Ryukyus, because the prevailing winds in this region are not toward these islands (see below). With this caution in mind, the trajectories of the SVP drifters are the best proxy to evaluate accidental drifts of small, primitive watercrafts.

Because the drift hypothesis for the Ryukyu Islands cannot apply to the northern migration routes that requires drifting against the Kuroshio, we focus on the southern migration route, where the ocean flow pattern is relatively simple and has been stable at least since the Last Interglacial Period. Small, low islands emerged above the sea surface off the coast of mainland China during the late Pleistocene (Fig. 1). We do not consider these islands as a start area of drifting, but behavior of the SVP drifters flowing from this area can be examined from the data for the southern migration route shown below.

## Backgrounds

### Geographic and archaeological settings

Taiwan and Luzon are sites where the Philippine Sea Plate is subducting beneath the Eurasian Plate. Their Pacific sides (east coasts) are characterized by the coastal or near-coastal mountains of ~ 1000–2000 m (Taiwan) or ~ 800–1600 m (Luzon) above sea level (ASL), fringed by the deep seas (Fig. 1).

The Ryukyu Islands Arc consists of ~ 100 small islands stretching over the area of 1200 km between Taiwan and the Kyushu Island of Japan. The arc is bordered by the Okinawa Trough in the East China Sea, and by the Ryukyu Trench in the Pacific. Three major geographic regions, the South, Central, and North Ryukyus, are defined.

The global sea-level 35,000–32,000 years ago is estimated to have been 70–80 m lower than today^20,21^. Figure 1 illustrates approximate geography of the region of the time, by lowering the sea-level 80 m using the modern seafloor topography. Most islands of the Ryukyu Arc preserve marine terraces that are considered to have been formed in the Last Interglacial Period^22^. With reference to the elevations of these terraces and supposing constant tectonic movements, the amounts of uplift of the Ryukyu Islands during the last 30,000 years are estimated to be 3–18 m (1–6 m/ky)^15^. Therefore, the islands in the Ryukyu Arc were probably slightly smaller than illustrated in Fig. 1.

A long-term isolation of these islands is also supported by neontological and palaeontological evidence^15^. For example, the extant fauna on these islands are endemic and lack major mammalian taxa such as monkeys and bears, which are common in both the Japanese main islands and Taiwan (except for the northernmost islands of Ryukyu which had been connected with Kyushu during the Last Glacial Maximum (LGM)); There were extinct, diminutive deer species on islands in Central and South Ryukyu until the late Pleistocene, suggesting “island rule” acting on these animals.

Palaeolithic sites, most of which are well-dated, are known from six different islands of the Ryukyu (Fig. 1). The excavated materials from these sites include marine shell tools, beads, as well as the world’s oldest fishhooks from Central Ryukyu^23^, and skeletal remains of anatomically modern humans from Central and South Ryukyu^24^. These collectively indicates sudden appearance of *H. sapiens* migrants in the Ryukyus both from north and south around 35,000 years ago, and the occupation of the entire arc by 30,000 years ago^15^.

### Kuroshio current in the present day

The Kuroshio is a strong western boundary current of the North Pacific subtropical gyre, which is ultimately controlled by the wind stress curl from the atmospheric circulation. This current, which transports warm and relatively high salinity water, originates off the Luzon coast, and flows poleward to develop into a fast (0.4–2.0 m/s), thick (< 1000 m), and wide (< 100 km) stream along the east coast of Taiwan^25,26^. Off northeastern Taiwan, the Kuroshio flows through the Yonaguni Depression and enters the East China Sea, bumps into the shelf-break of the East China Sea, and flows along it before turning eastward to exit to the Pacific through the Tokara Strait.

For the past decades, the Kuroshio has been intensively studied by various modern oceanographic methods including ship survey, moored current meters deployed in selected locations, experimental surface drifters, profiling floats that provides subsurface profiles up to 2000 m depth, and autonomous underwater gliders^25,27^.

Although the Kuroshio is relatively stable off the east coast of Taiwan and in the East China Sea, its velocity, width, position, and volume transport show some complex temporal and spatial variability^28,29^, with apparent timescales of about a week, ~ 100-days, and the season^25,30–32^ (Fig. 1). The mechanisms that cause such variations still remain controversial, but there are evidences that westward-propagating mesoscale eddies from the subtropical Pacific interact with the Kuroshio^28,29,31,33^. Some researchers suggested that monsoonal wind forcing also plays a certain role in affecting the Kuroshio’s path^32^. Statistically, the velocity core of the Kuroshio tends to shift landward in winter by strong northeast monsoon, and seaward in summer by southwest monsoon off Taiwan^30^. Whatever the actual causes are, the presence of temporal and spatial variation means that a drifted Palaeolithic watercraft would end up with different results depending on the timing of the accident.

In the onshore side of the Kuroshio, the coastal currents along the east coast of Taiwan are complex but are more modest in velocity and scale^28,29^. In the east and west of the main Kuroshio flow, ocean currents are relatively modest and varied with the impingement of mesoscale eddies.

### The Kuroshio during the last glacial period

The depth of the off the east coast of Taiwan and in the Okinawa Trough are ~ 1000–4000 m and ~ 1000–2000 m, respectively. At their junction, the topographic gap between Taiwan and the Yonaguni Island (the Yonaguni Depression) is shallower, but is still about 800 m deep. These bathymetric features constrain the flow path of the Kuroshio in the present day. Because such a topographic characteristic remained essentially unchanged since the late Pleistocene, there is no geographic ground to suppose distinct change of the Kuroshio's path, at least at its entrance to the East China Sea.

The continuing influx of the Kuroshio into the East China Sea through the Yonaguni Depression since the late Pleistocene is indicated by a number of studies on sediment cores and computer simulation modeling. Pollen of *Phyllocladus*, celery pines distributed in New Guinea and Southeast Asia, is reported in the LGM sediments obtained from northern East China Sea^34^. Sea surface temperature (SST) in the Okinawa Trough would be substantially colder if the Kuroshio did not enter there, but geochemically reconstructed SST consistently suggest that the temperature decrease in the trough during the Last Glacial Period was similar to the general trends in the tropical seas (− 3 to 5 °C)^35–42^. Ujiié et al.^42^ demonstrated that the SST in the Ryukyu Trench (outside the East China Sea) and Okinawa Trough (inside the East China Sea) behaved in a largely similar manner during the past 190 kyr. This supports that the ocean flow fields in this region have remained unchanged since the last interglacial.

Furthermore, according to a recent study on subsurface vertical temperature gradient in the Okinawa Trough, which serves as a direct indicator for the presence or absence of the Kuroshio, the reconstructed gradient during the LGM was similar to or smaller than today. This suggests that the Kuroshio in the Okinawa Trough was similar to or slightly stronger in the LGM than in the present day^43^. Finally, two simulation studies using 3-D numerical ocean models also concluded that the Pleistocene sea-level lowering did not change the course of the Kuroshio at its entrance to the East China Sea^39,44^.

Whether or not the volume transport of the Kuroshio was stronger, reduced, or stable since the late Pleistocene is debated, however. Many studies on foraminiferal fauna focus on *Pulleniatina obliquiloculata*, a warm water species commonly present in the modern Kuroshio. These studies report the decreases of this species in the Okinawa Trough during the LGM as well as in the mid-Holocene, which are called the “*Pulleniatina* Minimum Events (PME)”^35,45–51^. Some researchers interpret the PME as indicating times of weakened Kuroshio as a result of intensified winter monsoon and other factors^48^, but it may simply reflect the low winter SST^36,46,52^. Ujiié et al.^45,50^ used to propose that the PME was caused by the emergence of a land-bridge along the Yonaguni Depression, and resultant eastward deflection of the Kuroshio to flow into the Ryukyu Trench. However, this hypothesis does not explain the mid-Holocene PME, and sharply contradicts with the above-mentioned general uplifting trend in the region. Lin and colleagues reported that the mid-Holocene PME was not restricted to the East China Sea but widespread in the western North Pacific^53^. If this situation was similar in the late Pleistocene, the PME cannot be used as an indicator of the past Kuroshio.

Sea surface salinity (SSS) is also a controversial indicator for the past Kuroshio. The SSS at the Okinawa Trough during the LGM is reported either as lower than^37^ or similar^36,38^ to the present day. If the former was the case, the lower past SSS may reflect the weakening of the Kuroshio, influence from the terrestrial water in accordance with the eustatic continental expansion, precipitation, or mixture of these.

The two numerical studies which investigated the effect of sea-level change (but not other factors such as variation in winds) also reached contrasting results. One of them suggests reduced volume transport^44^, while the other increased maximum velocity (~ 25%)^39^ of the Kuroshio during the LGM. These studies also disagreed about the flow path of the Kuroshio in the Okinawa Trough. The former predicts southward shift of the location of the Kuroshio outflow from the East China Sea, whereas the latter concluded no such changes during the LGM. If the former was actually the case, the SST at the northern Okinawa Trough must have been 6–8 °C lower than today, but this is not supported by the geochemical proxy records cited above^39^.

In summary, the basic path of the Kuroshio remained unchanged during the past more than 100,000 years at least at its entrance to the East China Sea, and probably in further north. However, its intensity may or may not have been different from the present condition.

### Winds

Along the route of the Kuroshio from east of Luzon and Taiwan to the Ryukyu Islands, the prevailing wind is southwesterly monsoon (approximately in the direction of the Kuroshio) in summer, and northeasterly monsoon (against the direction of the Kuroshio) in winter. Westerly winds rarely occur and are blocked by the high mountains of Taiwan, if any. For example, at the Yonaguni Island located 110 km east from Taiwan, westerlies (winds from WNW–WSW) were observed less than 2% in the year of 2015, with average wind velocity 1.8 m/s excluding the influence of Typhoon (data from Japan Oceanographic Data Center). Because these high mountains had been formed well before the late Pleistocene, winds can hardly help Palaeolithic watercrafts to drift to the Ryukyu Islands.

## Results

**Figure 2:**
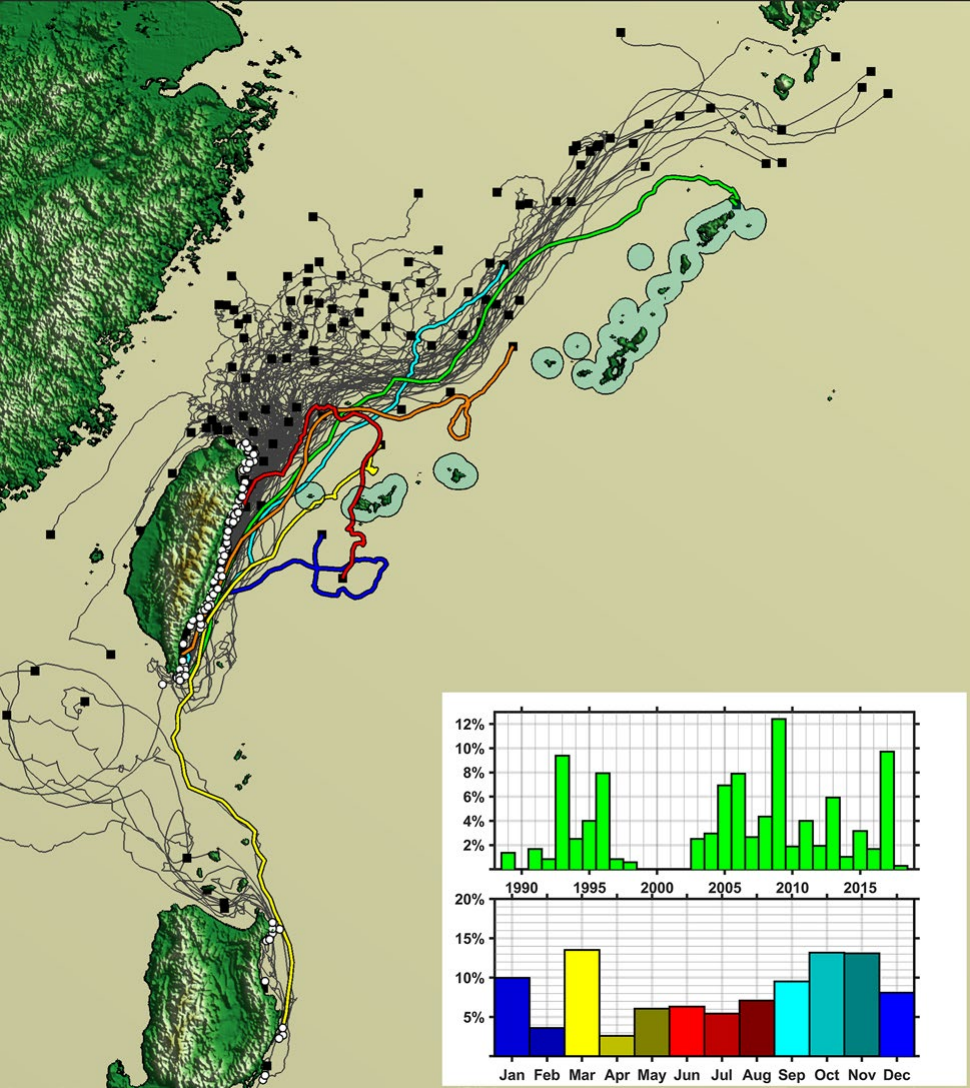
Trajectories of the 137 SVP drifters departed from off the east coasts of Taiwan and northern Luzon, and the years and the seasons of the track records. White circles and black squares are the start and end points of the drifters, respectively. Six trajectories that traversed the Kuroshio are colored. The areas within 20 km from the coasts of the islands of Central and South Ryukyus are circled (figure created using the Matlab R2019a: https://www.mathworks.com/products/matlab.html?s_tid=hp_products_matlab).

We identified 138 SVP drifters, which were released in or passed by the start areas off Taiwan (N = 122) and northeastern Luzon (N = 16). These can be regarded as a random sample from all seasons during 1989‒2017 (Fig. 2). Each of these drifters has a track record of 3–20 days.

Among the 122 buoys drifted from Taiwan, 114 were transported northward by the Kuroshio (here defined as the surface stream faster than 0.5 m/s) either from their start or partially on their ways. A drifting buoy must traverse the Kuroshio to approach the islands of Central and South Ryukyus, but most of the examined drifters (109/114) did not drift across the Kuroshio (Fig. 2, Supplementary Figs. S1–S3): 14 dropped off from it and were stuck in the coastal water of Taiwan; 87 ended up drifting on the shallow waters west of the Kuroshio, or were still moving on it inside the Okinawa Trough; 8 traveled more than 1000 km and approached one of the islands of North Ryukyu after 14–20 days of drifting.

**Figure 3:**
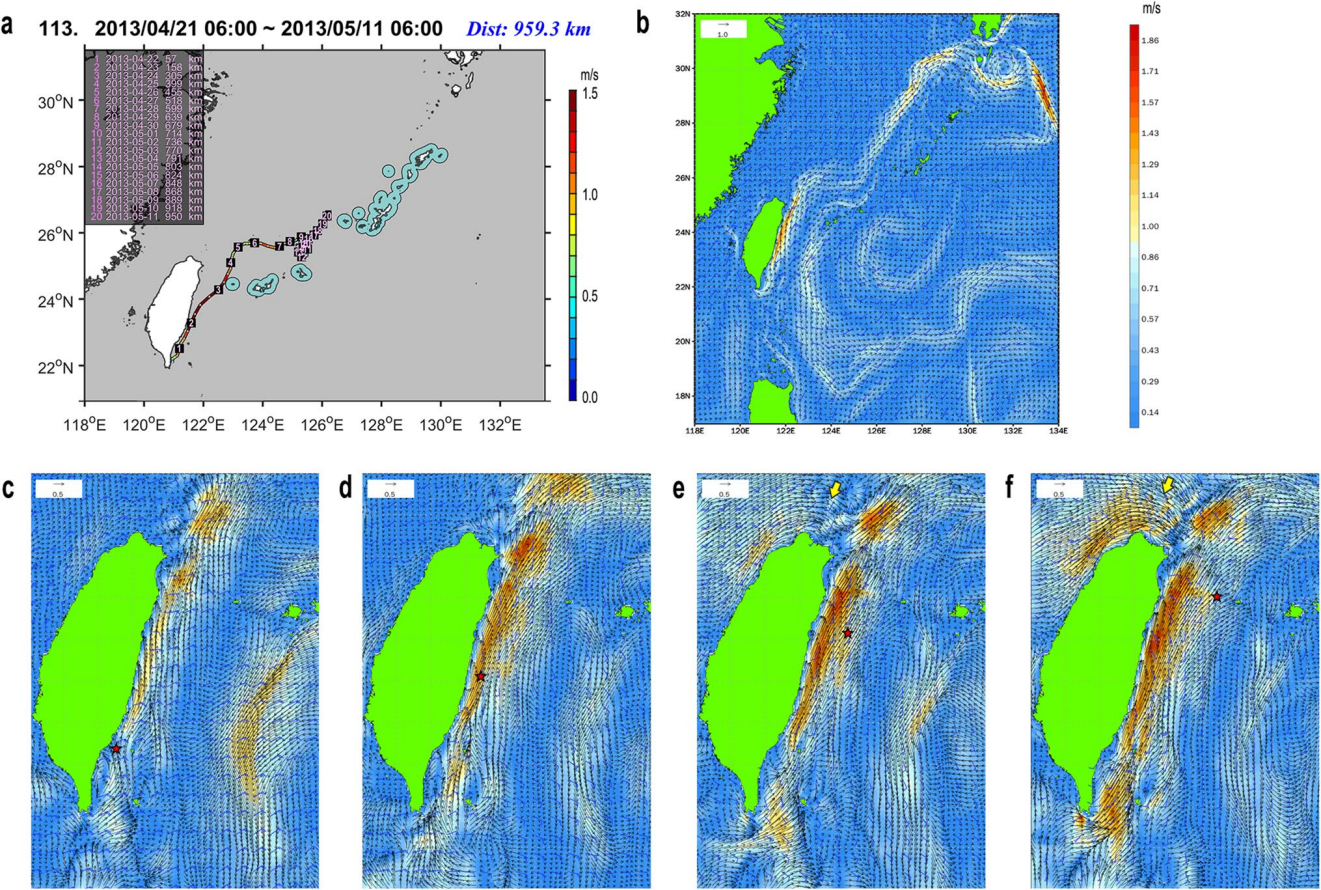
Trajectory of the SVP drifter No. 113 (ID: 114619, recorded in 2013: figure created using the Matlab R2019a) (**a**), and the ocean surface flows/winds reconstructed by the JCOPE-T (**b**‒**f**). (**b**) Daily mean for the fourth day. (**c**‒**f**) hourly mean with the location of the drifter (star). Wind direction and speed are indicated by the frag-like symbols with feathers and half-feathers representing 10 and 5 knots, respectively. The Kuroshio was weak overall under strong NE winds (~ 10 m/s). After passing through the weak and narrow stream of the Kuroshio off southern Taiwan on the second day ((**c**), 6:00 of April 22), the drifter shifted toward the offshore side of the Kuroshio ((**d**), 0:00 of April 23). On the third day ((**e**), 18:00 of April 23), the Kuroshio main axis slightly inclined east under the impact from the eastward flow emerged in the north of Taiwan (yellow arrow), and the drifter was brought further offshore in this condition, to pass near the Yonaguni Island on the fourth day ((**f**), 6:00 of April 24). It then entered the East China Sea, and was dropped off to the south of the Kuroshio on the tenth day to experience circular drifting without effectively approaching to any islands.

**Figure 4:**
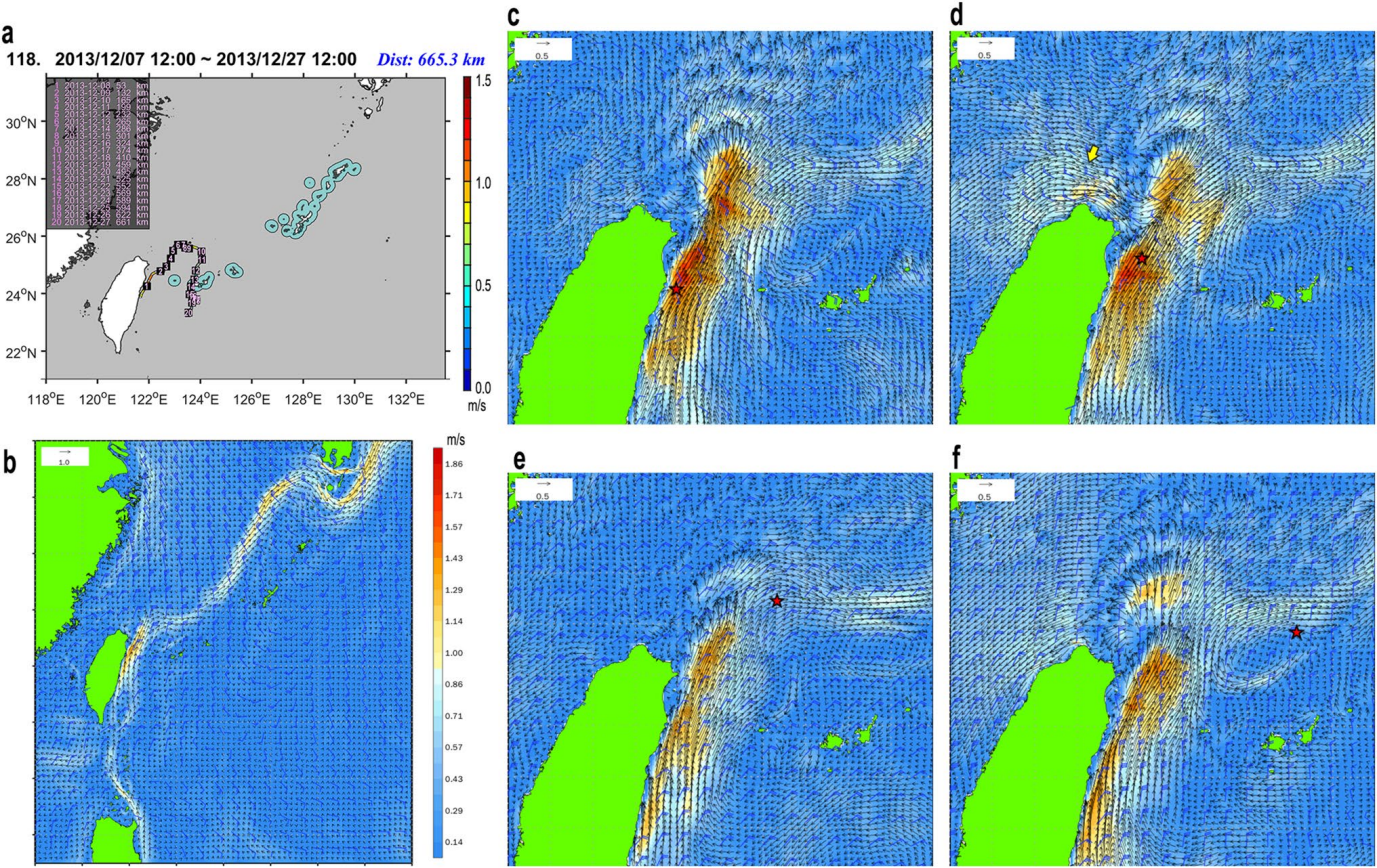
Trajectory of the SVP drifter No. 118 (ID: 114620, recorded in 2013; figure created using the Matlab R2019a) (**a**) and the ocean surface flows/winds reconstructed by the JCOPE-T (**b**‒**f**). (**b**) Daily mean for the second day. (**c**‒**f**) hourly mean with the location of the drifter (star). Wind direction and speed are indicated by the frag-like symbols with feathers and half-feathers representing 10 and 5 knots, respectively. The Kuroshio was strong off northern Taiwan on the first day ((**c**), 12:00 of December 8). Strong easterly (~ 8 m/s) for the first 2 days changed to very strong NW‒N winds (~ 13‒5 m/s) after 0:00 on December 9 (**d**), when the eastward flow emerged in the north of Taiwan (yellow arrow) and the drifter deflected slightly eastward toward the offshore side of the Kuroshio. Then, after traveling north ((**e**), 12:00 of December 13) and then east along the weakened Kuroshio flow, on the tenth day ((**f**), 12:00 of December 17), the drifter was dropped off to the south of the Kuroshio. It then slowly moved south to pass near the Iriomote Island, under the continuing strong northerly (~ 10 m/s).

Five drifters traversed the Kuroshio to reach its offshore side, and further drifted the areas either distant from or near (< 20 km) the islands in Central and South Ryukyus (Fig. 2). High-accuracy, hourly current/wind data based on the JCOPE-T are available for two of them to inspect the detailed background of their movements. Figures 3 and 4 indicate that the both cases occurred under strong winter monsoon (northeasterly), and when the Kuroshio flow was weakened, disturbed, or altered. Additionally, submesoscale and mesoscale eddies rotating either clockwise (anti-cyclonically) or counter-clockwise (cyclonically) which impinge onto the Kuroshio from the east could potentially make the drifter moving across the main stream of the Kuroshio.

**Figure 5:**
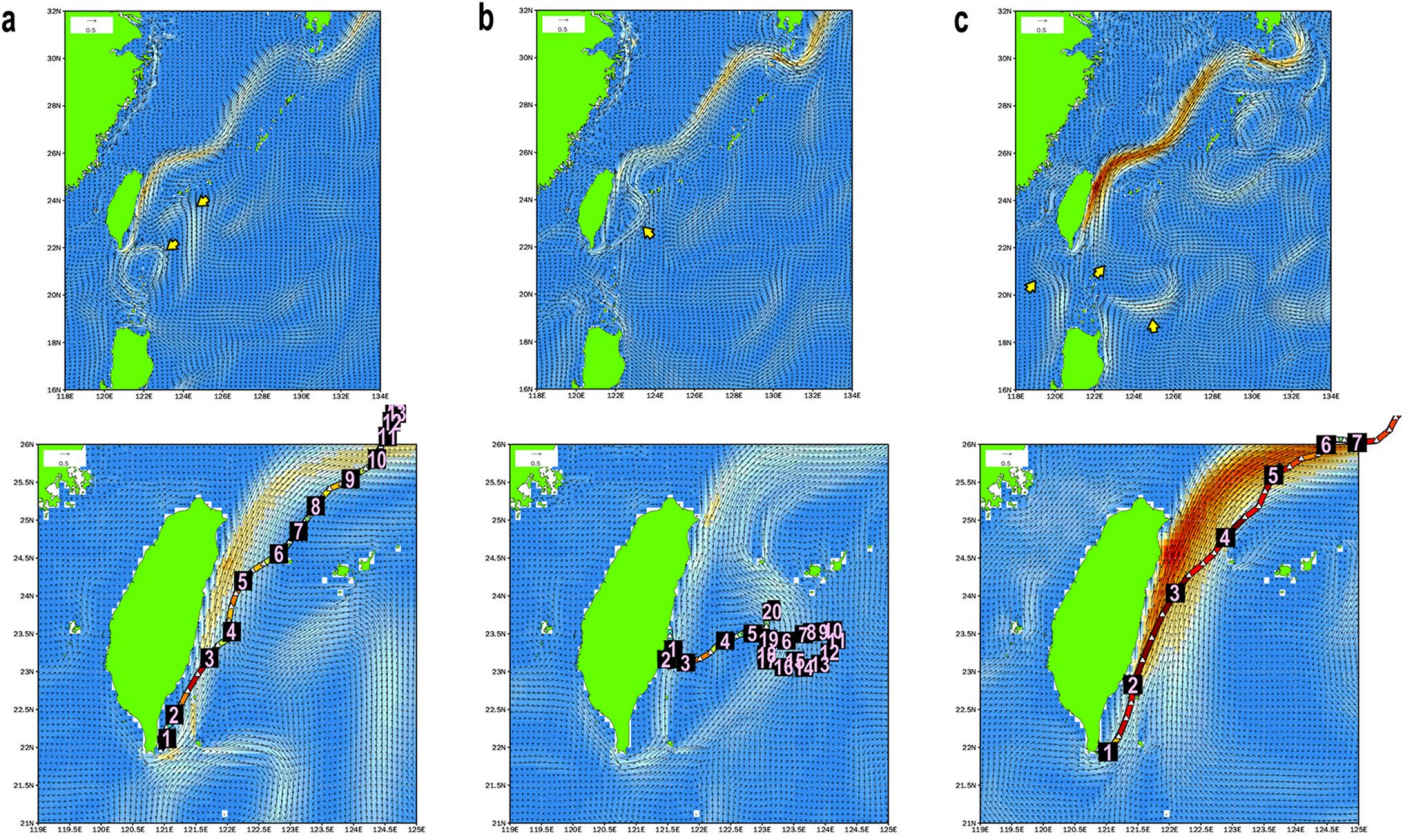
Trajectories of three SVP drifter that traversed the Kuroshio superimposed on daily averaged ocean surface flows reconstructed by the JCOPE2. (**a**) Drifter No. 15 (ID: 9320606) mapped on the currents of the fourth day, October 23, 1993. (**b**) Drifter No. 14 (ID: 9320602) on the currents of the third day, November 13, 1993. (**c**) Drifter No. 67 (ID: 62304) on the currents of the fourth day May 27, 2006. The yellow arrows indicate mesoscale eddies which may affect the strength and pattern of the Kuroshio. See Fig. 3 for color scales. See Supplementary Figs. S1–S3 for more information.

Lower-resolution, daily mean current data based on the JCOPE2 are available for the other three cases. Two of them were again associated with winter monsoon, and the Kuroshio was weakened with the impact from clockwise and counter-clockwise mesoscale eddies (Fig. 5a,b, Supplementary Figs. S4, S5). The other case occurred when the Kuroshio is strong (Fig. 5c, Supplementary Fig. S3). How this drifter traversed the strong current is uncertain with the available current data, but we infer that one of the key events was at its start from the southern tip of Taiwan. On this day, the Kuroshio was disrupted in the south of Taiwan because of complex interaction with mesoscale eddies, and the temporally emerged eastward stream brought the drifter offshore (Supplementary Fig. S6).

**Figure 6:**
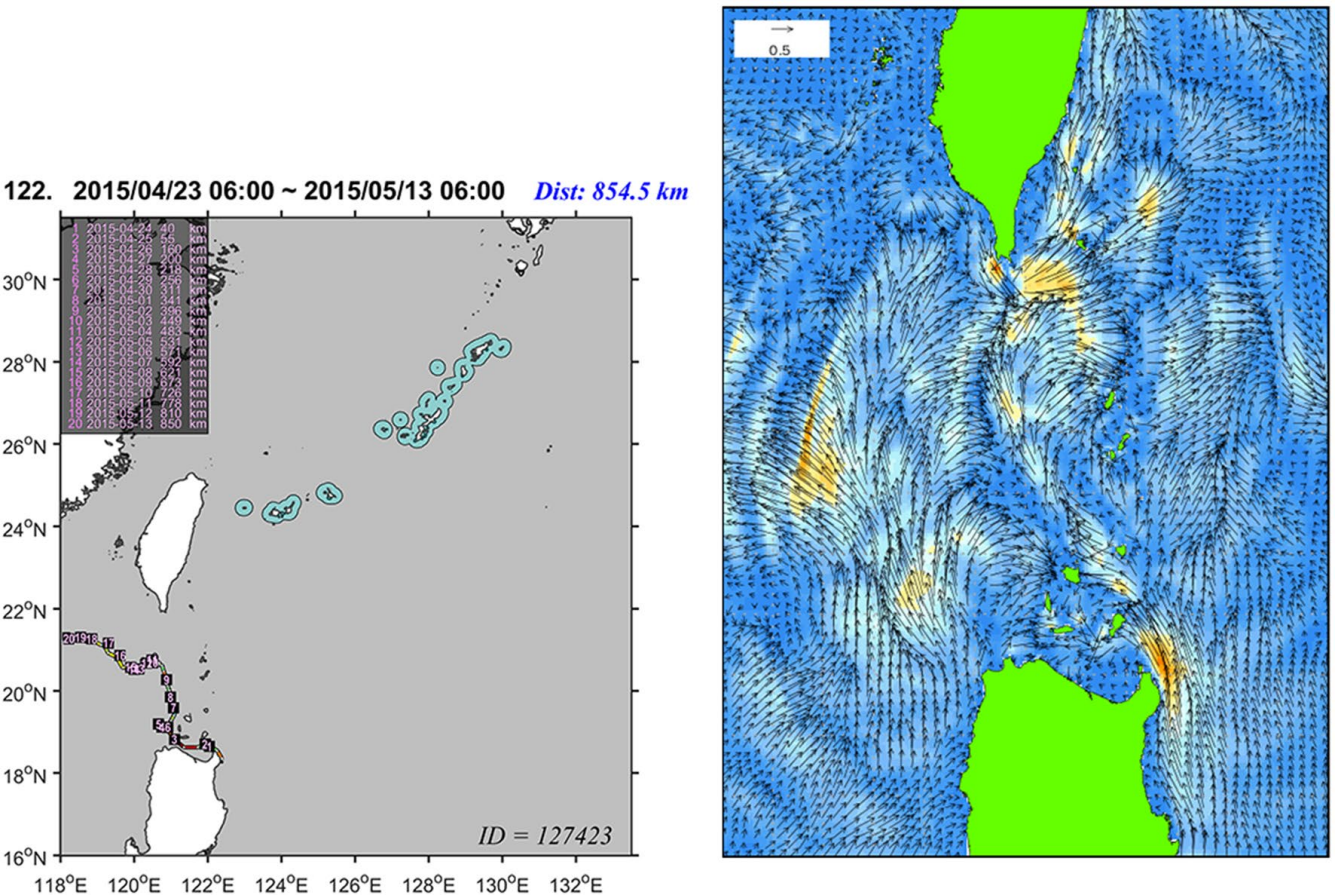
An example of the buoy drifted from Luzon to the South China Sea (No. 122, ID:127,423, recorded in 2015; figure created using the Matlab R2019a). The trajectory (left) and hourly ocean surface flow on its first day (12:00 of April 23) reconstructed by JCOPE-T. Note that the Kuroshio at the northeastern corner of Luzon branches off toward the South China Sea at multiple locations. These streams transport many of the drifters departed from Luzon westward.

Among the 16 buoys drifted from northern Luzon, 13 were trapped by the Kuroshio somewhere on their ways. Most of them (10/13) moved northwest to finally drift around the Babuyan Islands or in the South China Sea, and a few (2/13) stayed in the coastal water of Luzon after 10–12 days of drift (Fig. 2, Supplementary Figs. S1–S3). The ocean flow fields reconstructed by the JCOPE series indicate that such movements are because the coastal stream of the Kuroshio off Luzon usually spill out toward the South China Sea in the north of Luzon (Fig. 6). The single exception in our observation was the buoy drifted in June 2011, which was transported to the South Ryukyus and passed near the Yonaguni Island after 13 days of drift. This was most likely a consequence of a typhoon (Supplementary Fig. S7).

## Discussion

The above analyses demonstrate that a floating object drifted from off Taiwan by the Kuroshio has a certain chance to approach islands in North Ryukyu after more than 14 days (8/114 or 7% in the case of our sample of the SVP drifters). However, such an object rarely traversed the Kuroshio (5/114 or 4%), and has a limited chance to get close (< 20 km) to the Central and South Ryukyus (3/114 or 3%). Figure 2 also demonstrates that there would be almost no chance for a small boat drifted from one of the low islands, which had been exposed off the mainland China during the late Pleistocene (Fig. 1), to reach the Pacific side of the Kuroshio. Among the available small sample of the buoys drifted with the Kuroshio from Luzon (N = 13), only one moved toward the Ryukyus under the influence from a typhoon. The situation must have been similar in the late Pleistocene, because the flow path of the Kuroshio has been unchanged in this region since then (see above).

There are some reasons to believe that the actual probability for a drifted Palaeolithic watercraft to reach the Central and South Ryukyus was less than the statics obtained from the SVP drifters. First, compared to a watercraft of any type, a SVP drifter receives less force from the local monsoonal wind, which pushes a floating object away from the target islands in summer. Second, four of the five SVP drifters traversed the Kuroshio under moderate to strong northeasterly winter monsoon. Conceivably, people would not go to sea in such a condition, because the sea surface becomes choppy when current and wind contradict to each other. Winter was the off-season of marine fishing for the Amis people native to Taiwan, who used to fish using a sea-going rowing bamboo raft. Third, the drifters from Luzon always pass through the Babuyan (and Batanes) Islands, suggesting that most drifted watercrafts would arrive at these islands without traveling further toward Taiwan and the Ryukyus.

In addition, accidental arrival and accidental migration are related but different concepts. The latter, the objective of this study, depends on aspects of the group arrived on the island, such as the number, age and sex composition, mortality, fertility, and other factors relevant to the later population growth. This initial group size and composition depends on (1) the frequency of past drift accident, (2) the chance of arrival of the drifted crafts to the island, and (3) the number and composition of the people on board. Our analyses described above indicated low chance for the second factor in the Ryukyu region, but the ultimate success probability of migration by accidental drift must be evaluated in this whole framework.

There is a controversy if the viable initial group size is large (> 1300 individuals)^9^ or small (a few tens or less)^54^. Ihara et al.^55^ recently demonstrated that this group size depends heavily on fertility and mortality levels, which are unknown for the past populations, but a group of ten or fewer unrelated young men and women can be successful within the range feasible for recent hunter-gatherers.

Ihara and colleagues also argued that, in relation to the third point of the above, prehistoric hunter-gatherers who traveled on a boat, or jointly on multiple boats, were unlikely to be a free mixture of males and females, but probably consisted of closely related individuals such as households. This is because various ethnographic documentations inform that a travel of hunter-gatherers occurs basically with a single family or multiple families as a unit, either on land or at sea. It should be noted here that, if there was gendered division of labor in fishing and men and women did not share the same fishing boat as exemplified in hunter-gatherers’ communities in Australia^56^ as well as in Amis farmers in Taiwan^57^, accidental drift of fishing boats does not lead to migration because of the completely biased sex composition.

Taken together, Palaeolithic accidental drift may lead to a successful migration when more than two families landed on the same island (or island cluster) almost at the same time, either (A) separately by different crafts, or (B) with a large craft shared by multiple families.

Our examination of the SVP drifters demonstrated that the case A is virtually impossible for the Ryukyu Islands; the case B is also difficult but could occur if such sharing of a large craft was common, and if such crafts were frequently entangled in accidental drift. The latter factors can only be speculated for the populations in deep past, but at least repeated occurrence of accidental drift seems unrealistic. This is because people could choose a calm day for safe sea travel; they should have brought spare paddles to reduce a risk of drift; and there is no reason for a large craft shared by multiple families to go offshore. A small and mobile fishing boat may want to head out to sea to find a good fishing place, but traveling families would choose nearshore water course for safety. When the crew detected a danger, they must have tried desperately to return to Taiwan or Luzon, which can easily be found from offshore because of their high elevations. Actually, the Amis fishermen were well-acquainted with the limitation of their rafts and hardly ran a risk at sea. According to our interview to Amis elders at the Makrahay Village in Taitung County, eastern Taiwan, they have never encountered or heard about marine accidents of their traditional bamboo rafts.

**Figure 7:**
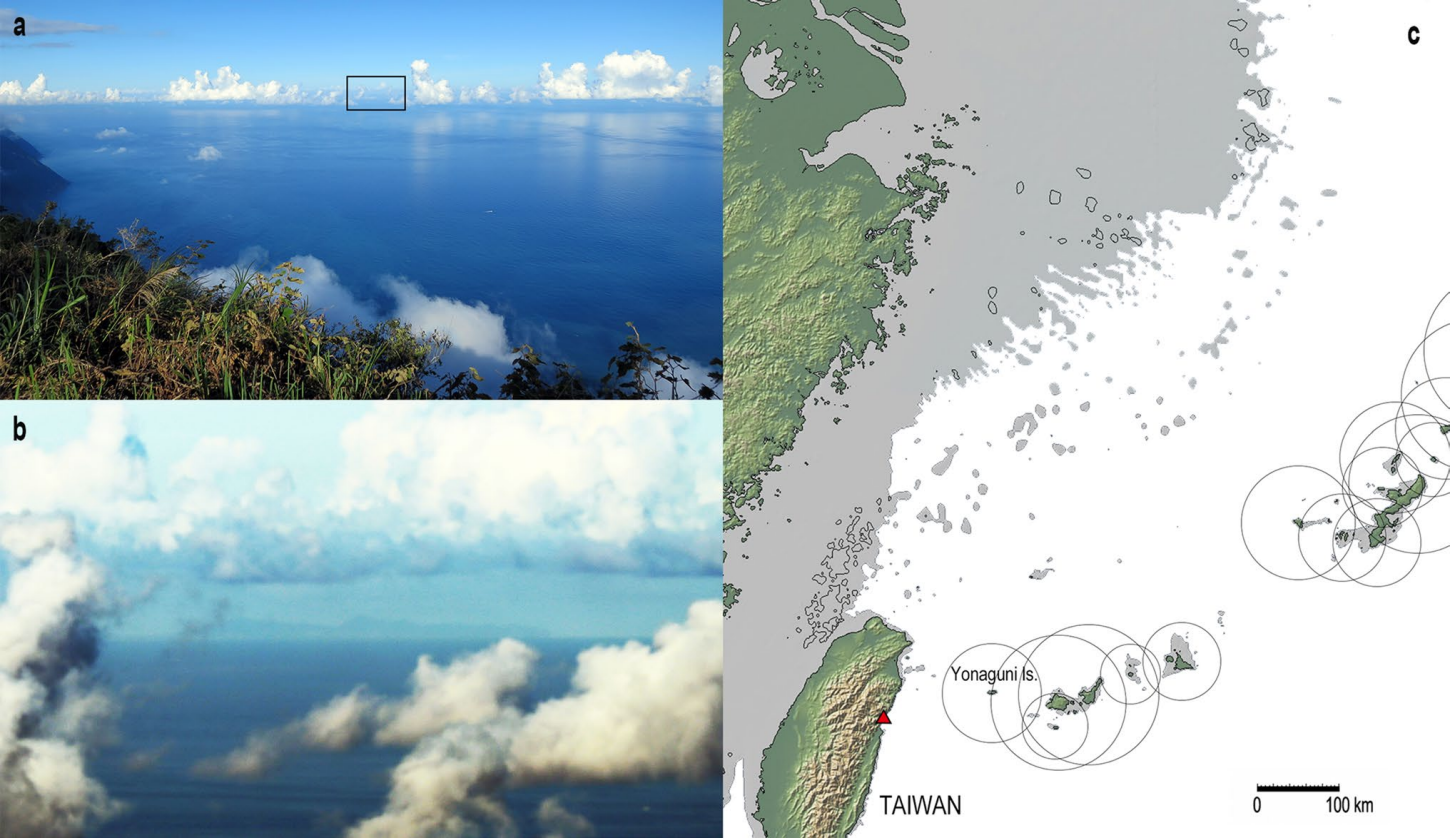
(**a**) View from a coastal mountain of Taiwan (Liwushan, Hualien County: about 1200 m ASL). (**b**) Yonaguni Island seen at the square in A. This island is rarely visible. It may be seen in the evening with slanting sunlight from the back (as this photo), or as a silhouette in front of the rising sun just before the dawn, when the sky is very clear with few clouds around the horizon. (all photographs by Yousuke Kaifu on August 27, 2017). (**c**) Estimated palaeogeography 35,000–32,000 years ago when the sea level was 80 m lower than today, with the maximum visibilities of the islands of southern Ryukyu from sea surface (circles, see “Methods” section) (map created using the GeoMapApp 3.6.10 software based on the Global Multi-Resolution Topography synthesis^64^). The viewpoint for (**a**,**b**) is indicated by the red triangle.

Our results support that late Palaeolithic people set out for the islands of South and Central Ryukyus deliberately about 35,000 years ago, to cross the strong current and head for the island which was invisible beyond the horizon (Figs. 1, 7). In the western Pacific, there is evidence for earlier, extensive colonization of islands by 47,000 years ago (Wallacea and Sahul)^19^, advanced fishing technology dated to 42,000 years ago (Timor Island^8^), and the world’s oldest round voyage to acquire exotic materials as early as 38,000 years ago (Central Japan)^58^. The evidence reported here from another area of the region signals that such maritime activities were expanding and growing during the Marine Isotope Stage 3, as people’s active exploitation of new environments associated with some technological advancement such as fishhooks^8,23^ and new watercrafts^59^.

Finally, we note that not a small number of the SVP drifters departed from off Taiwan returned home or flowed back toward the continent (Fig. 2). This suggests that a drifted Palaeolithic watercraft had a certain chance to return alive, and people could accumulate knowledge about the offshore current through such experience. This is particularly likely because Taiwan is easy to find from offshore due to its high elevation up to 3952 m ASL. The Yonaguni Island, the nearest island of the Ryukyus, is not visible from the coast of Taiwan, but is occasionally visible from its coastal mountains (Fig. 7). We speculate that these circumstances fostered the idea and some practical plan for the local Palaeolithic people to migrate to this remote island about 35,000 years ago.

## Methods

### SVP drifter

The SVP drifter is designed to monitor surface current velocity, water temperature, and other marine environmental proxies in Lagrangian frame of reference^60,61^. The positions of these freely drifting buoys were updated hourly via the Argos satellite system. After undergone strict processing and correction procedure by National Oceanic and Atmosphere Association (NOAA), quality-assured geographical positions of SVP drifters are released at 6-h interval^62^. Each SVP drifter has a spherical surface float of 30.5–40 cm diameter, and is equipped with a drogue centered at 15 m beneath the surface to follow the surface current. It moves by a combined effect from average upper-ocean current at 15 m depth and wind-driven surface flow (also called “Ekman drift”), but is designed to minimize the “slip” by wind. We extracted the maximum of 20 days of each track record from its start off Taiwan and Luzon.

### Start areas

We define the location to start drift as within 10 km from the east coasts of Taiwan or northern Luzon, with the assumption that a Palaeolithic watercraft usually operated fishing or transport within these areas. This seems reasonable because the speed of a rowing bamboo-raft would not exceed 3 km/h^59^, and it takes more than 3 h to go 10 km offshore with such a craft. Because an underlying assumption of the drift hypothesis is that Palaeolithic people did not have enough skills to cross the fast-flowing major ocean current like Kuroshio, we exclude the two islands off the southeastern Taiwan (Green Island, and Orchid Island) as starting areas, which are located more than 30–60 km offshore and in the midst of the Kuroshio.

### Numerical modeling of ocean current

We interpret the movement of each SVP drifter with reference to ocean surface flow fields reconstructed by supercomputer-based ocean current simulator called JCOPE2 (track records of 1993–2012) and JCOPE-T (track records of 2012–2017). The JCOPE (abbreviation of the Japan Coastal Ocean Predictability Experiment) series is developed by the Japan Agency for Marine Earth Sciences and Technology (https://www.jamstec.go.jp/jcope/vwp/). They use observation data of sea-surface elevation, temperature and salinity, together with data on wind, heat fluxes, and tidal forces (JCOPE-T only) to calculate and visualize the ocean-current conditions on daily (JCOPE2) or hourly (JCOPE-T) bases, with the resolution of 9 km (JCOPE2) and 3 km (JCOPE-T), with high accuracy (JCOPE-T in particular). See ref.^63^ for more information about JCOPE-T.

### Island visibility during voyage

The target island becomes visible above the horizon only within a certain distance, during the daytime when the sky is clear. To illustrate Fig. 7c, we calculated such a maximum distance as the distance from the summit of the island to the geometric horizon, using the following formula^12^:

$$ {\text{Visibility }}\left( {{\text{km}}} \right) \, = { 3}.{57 } \times {\text{ SQRT }}\left( {{\text{h}} + {8}0} \right) $$

where h (in meter) is the height of the island in the present day. We added 80 m to the height in consideration of the sea-level lowering at the time of initial migration. This formula does not consider several factors that increase or decrease the distance. The former includes the eye height of the voyager and refraction in the atmosphere. The latter includes estimated uplift of the islands of Ryukyus during the last 35,000 years (~ 10 m)^15^, and the fact that several tens of meters of the island has to be above the horizon to find a remote island at sea.

## Supplementary information

Supplementary Information.
