# Supplementary material for: Palaeolithic voyage for invisible islands beyond the horizon
Source: Sci Rep. 2020 Dec 3;10:19785. doi: 10.1038/s41598-020-76831-7 (PMC7714783; doi:10.1038/s41598-020-76831-7)
Supplement: Supplementary file 1 — Supplementary Information. [file 41598_2020_76831_MOESM1_ESM.pdf]

Supplementary Information

for

Palaeolithic voyage for invisible islands beyond the horizon

Yousuke Kaifu, Tien-Hsia Kuo, Yoshimi Kubota, Sen Jan

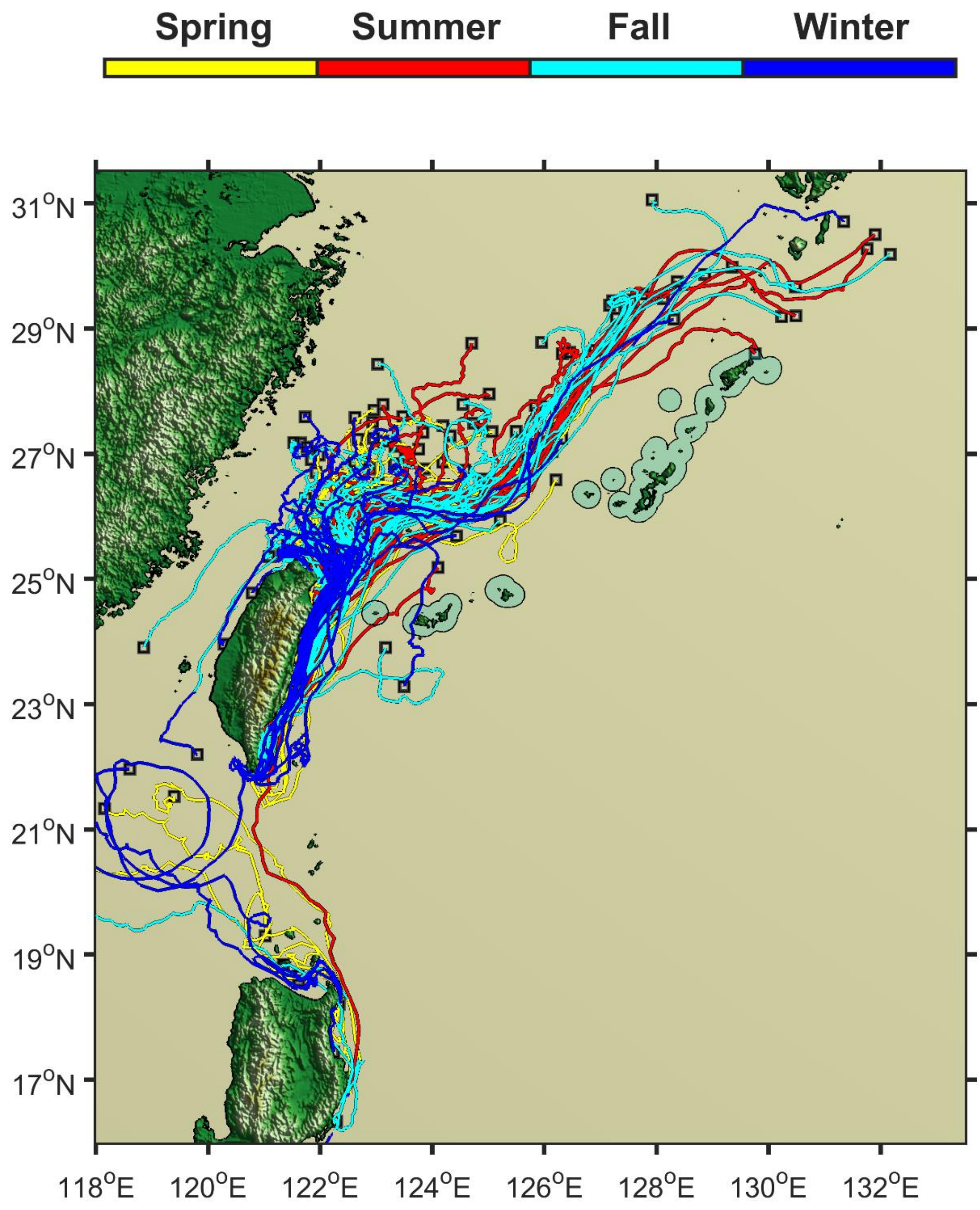

Supplementary Fig. 1. Trajectories of the 137 SVP drifters sorted by the recorded seasons (figure created using the Matlab R2019a: https://www.mathworks.com/products/matlab.html?s_tid=hp_products_matlab).

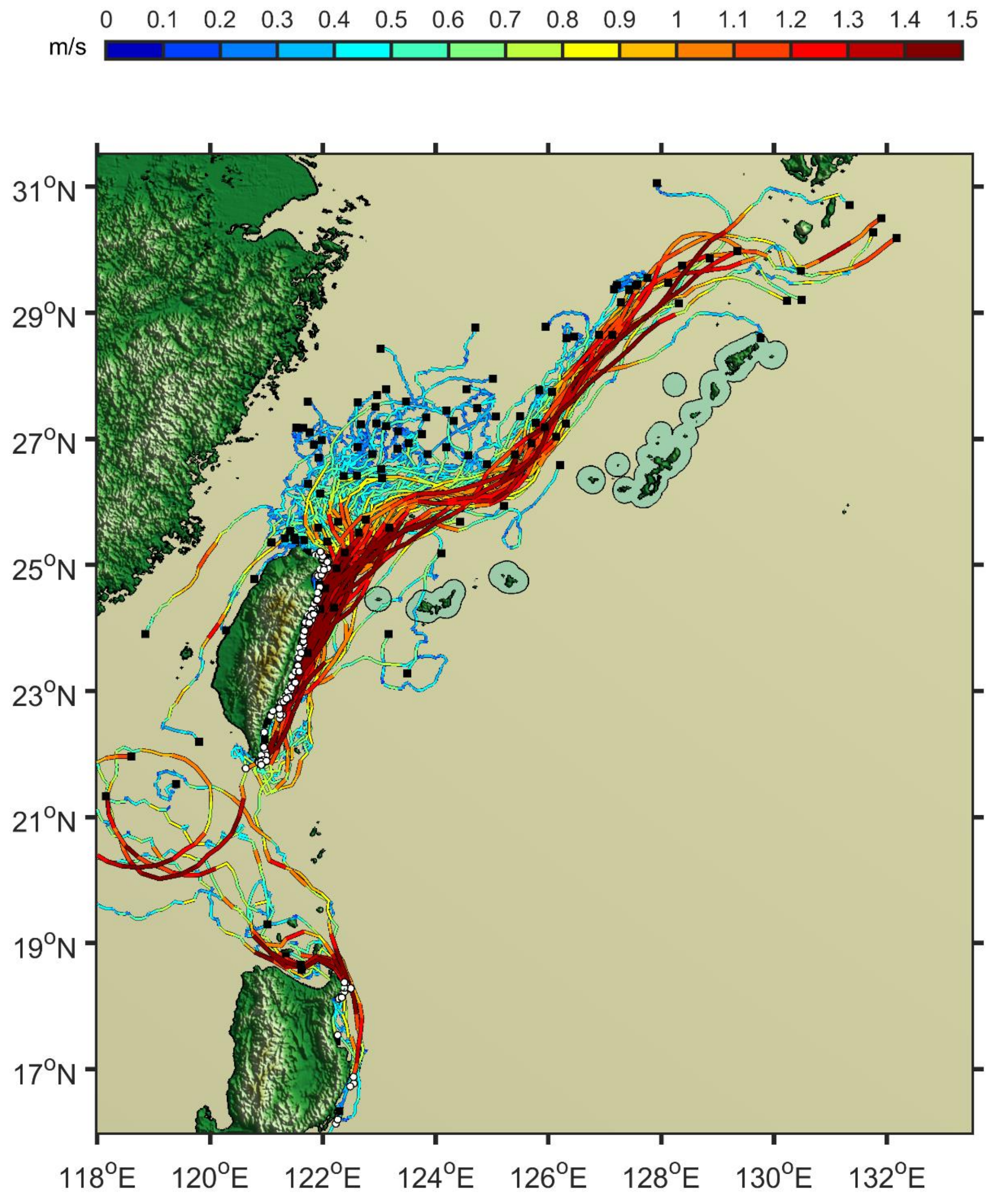

Supplementary Fig. 2. Trajectories of the 137 SVP drifters colored by the speed (figure created using the Matlab R2019a).

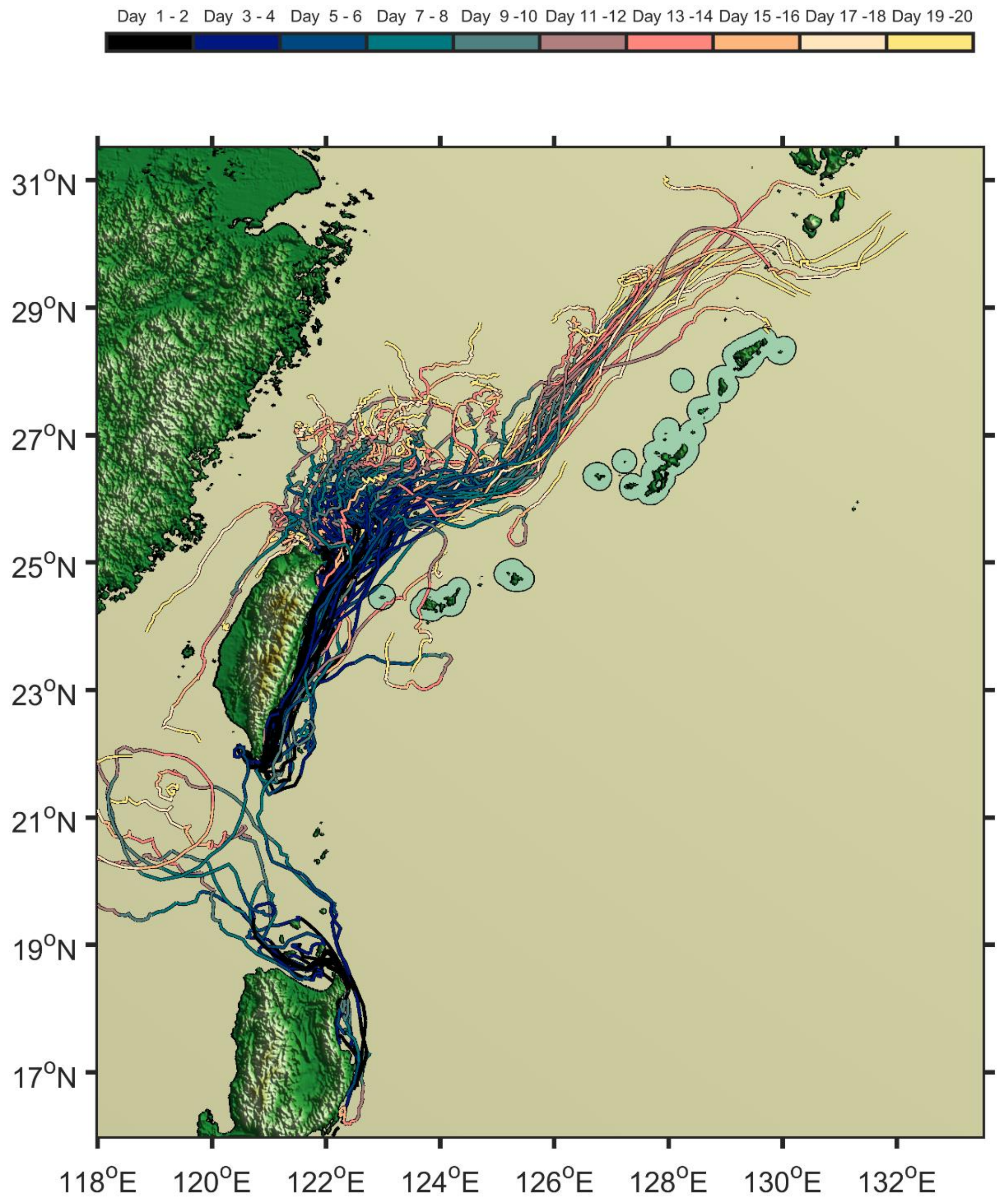

Supplementary Fig. 3. Trajectories of the 137 SVP drifters colored by the number of days of the drift (figure created using the Matlab R2019a).

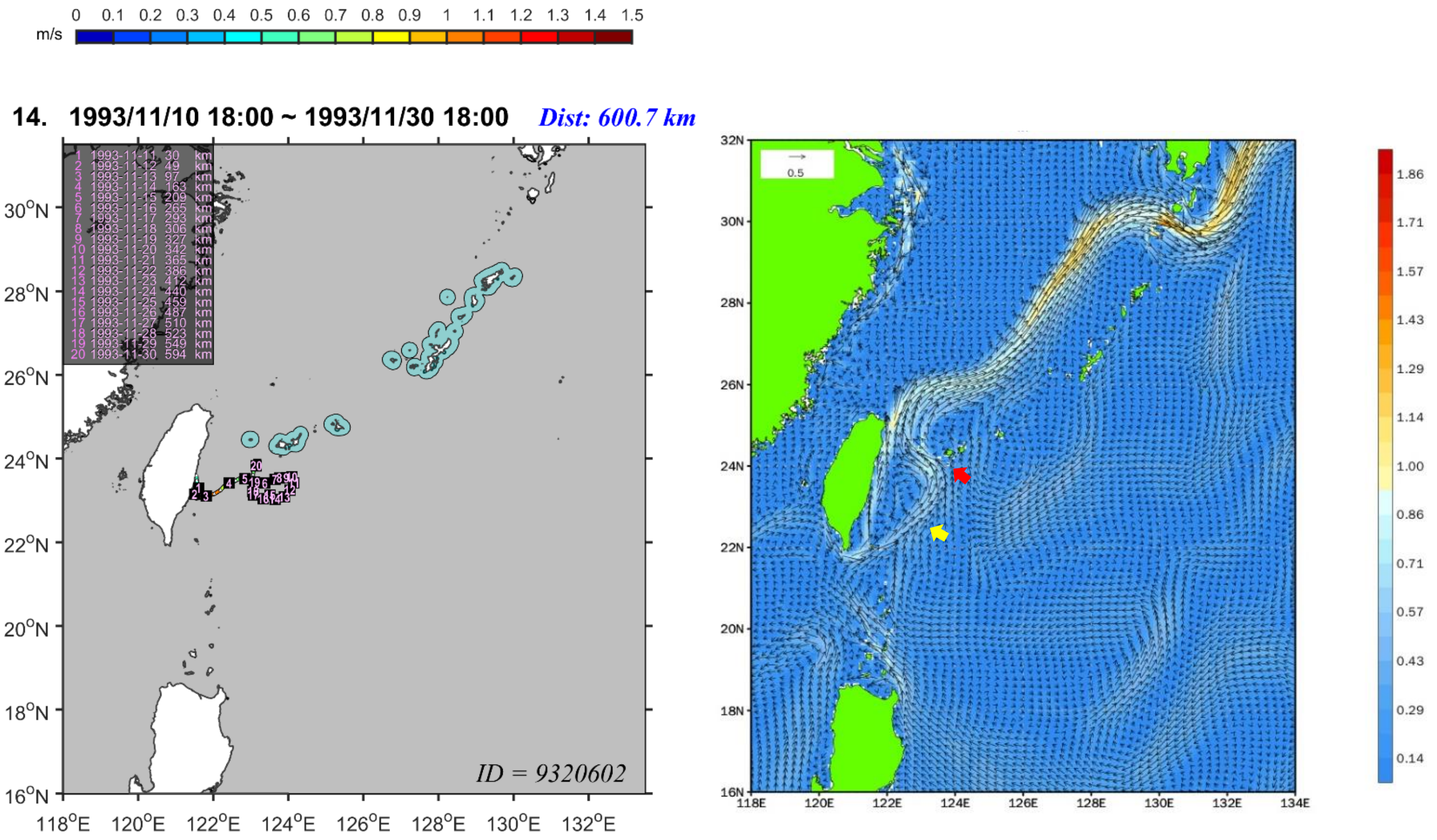

Supplementary Fig. 4. Trajectory of SVP drifter No. 14 (left, created using the Matlab R2019a) and the ocean surface flows on its third day (right: daily average for November 13, 1993, reconstructed by JCOPE2). In the right panel, the Kuroshio is narrow and very weak off Taiwan, under the impact from a counter-clockwise mesoscale eddy (yellow arrow) and moderate upwind (NE 6.0−4.0 m/s, hourly data for November 13 measured at the Hateruma Island (red arrow) by the Japan Meteorological Agency). In this circumstance, the buoy was not transported north but drifted eastward to cross the Kuroshio. It then moved further offshore and drifted in a circular manner without approaching any islands until the 20th day.

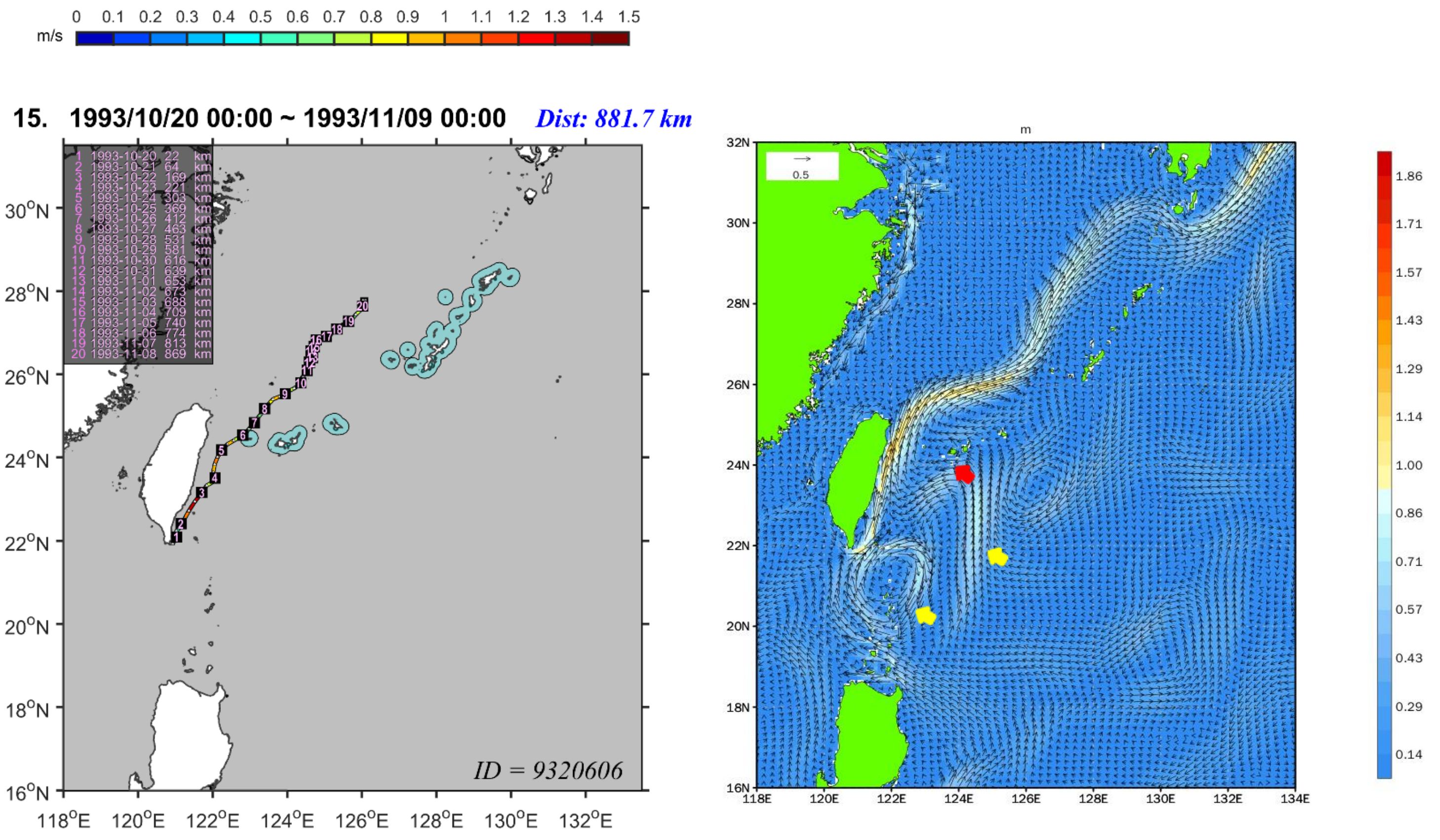

Supplementary Fig. 5. Trajectory of SVP drifter No. 15 (left, created using the Matlab R2019a) and the ocean surface flows on its fourth day (right: daily average for October 23, 1993, reconstructed by JCOPE2). The buoy shifted eastward to reach the Pacific side of the Kuroshio on this day (the track between “3” and “4” in the left panel), traversing the narrow and weak Kuroshio off South Taiwan, which was impacted by mesoscale eddies (yellow arrows) and relatively strong northerly (hourly averages were NNE−NEE 7.0−4.0 m/s during the initial four days, measured at the Hateruma Island (red arrow): Data from Japan Meteorological Agency). The buoy then moved northeastward along the Pacific side of the Kuroshio to pass near the Yonaguni Island on the sixth day.

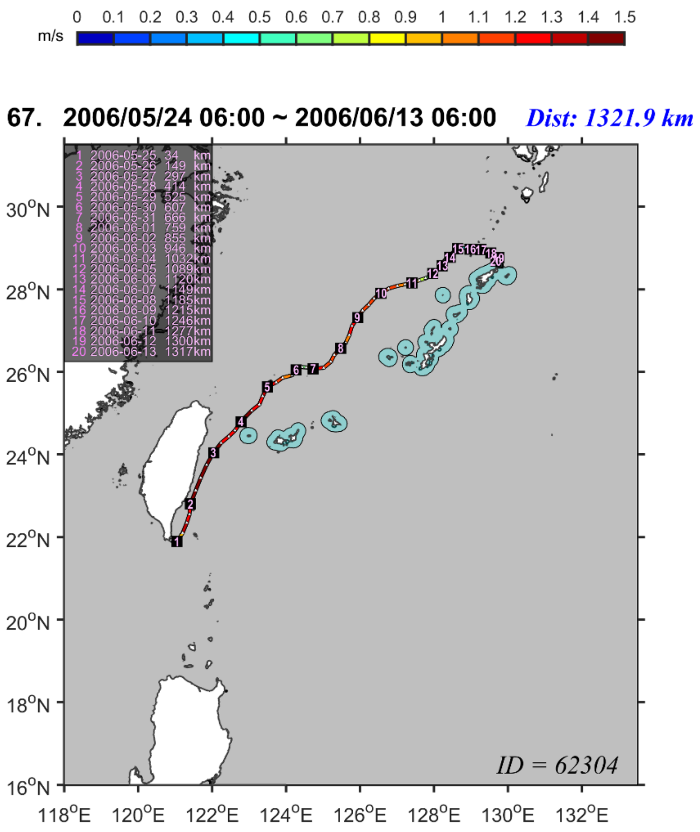

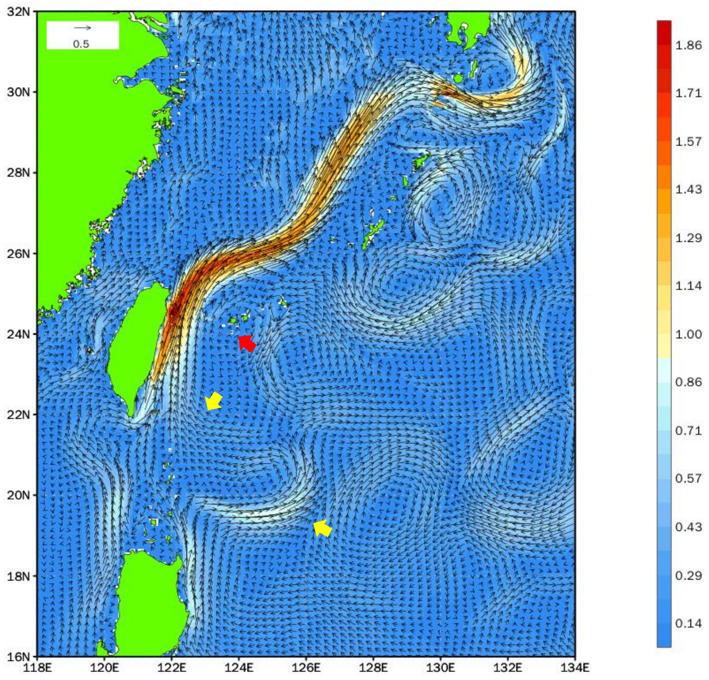

Supplementary Fig. 6. Trajectory of SVP drifter No. 67 (left, created using the Matlab R2019a) and the ocean surface flows on its first day (right: daily average for May 24, 2006, reconstructed by JCOPE2). The Kuroshio was strong at this time, but a counter-clockwise and a clockwise mesoscale eddies (yellow arrows) impacted and interrupted the Kuroshio flow between Luzon and Taiwan, and a relatively strong eastward stream emerged at the southern tip of Taiwan. This stream transported the buoy offshore to drift north along the Pacific side of the Kuroshio, to finally approach the Amami-Oshima Island after 20 days of drift over 1322 km. Northerlies and southerlies alternated during this period (data observed at the Hateruma Island by the Japan Meteorological Agency).

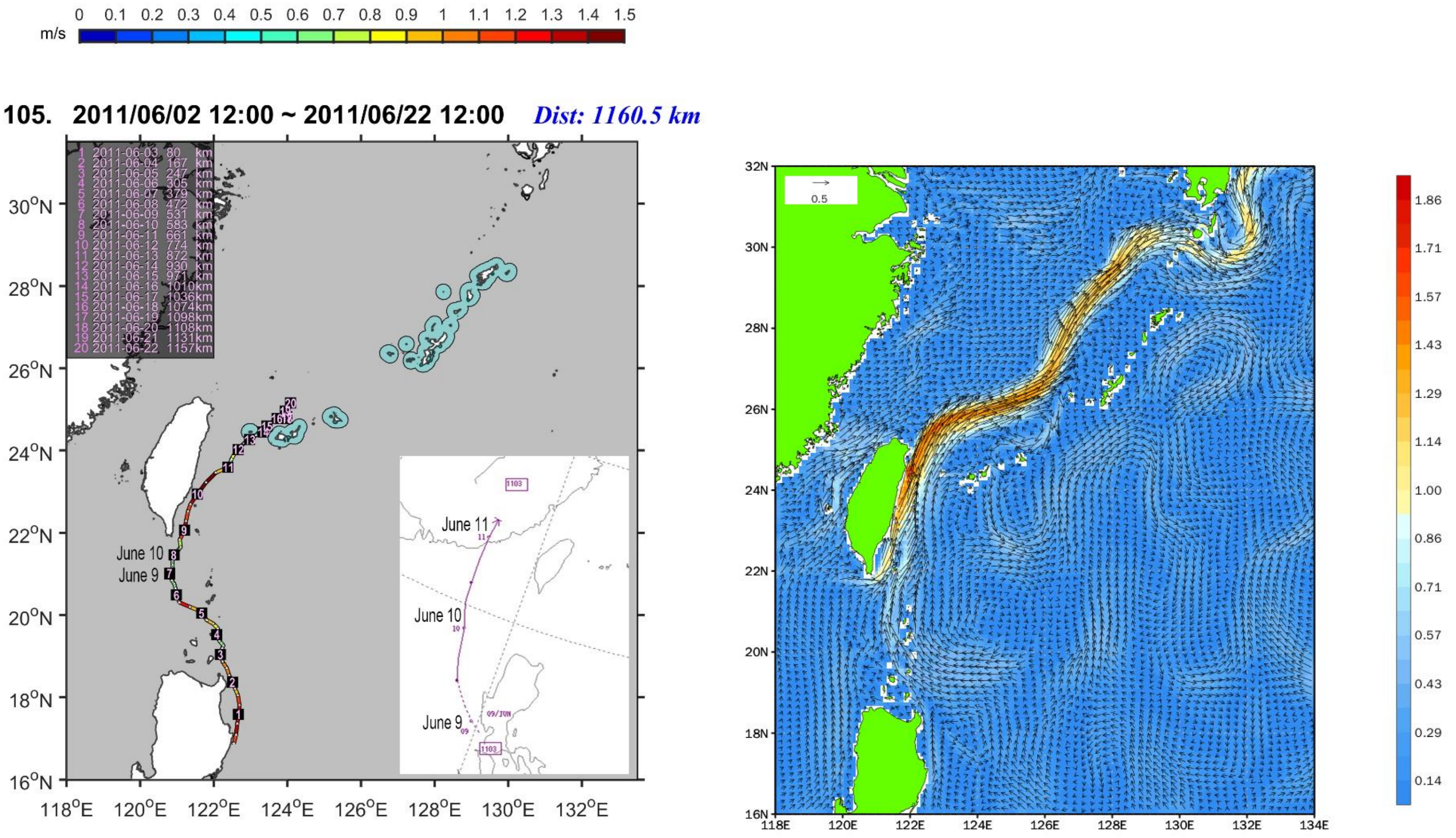

Supplementary Fig. 7. Trajectories of the SVP drifter No. 105 (ID = 82411, created using the Matlab R2019a) and a typhoon (left, data of the typhoon from the Japan Meteorological Agency: https://www.data.jma.go.jp/fcd/yoho/typhoon/index.html), and the ocean surface flow on June 9, 2011 (right, daily mean reconstructed by the JCOPE2). The northward deflection of this buoy on June 9 in the middle of the Luzon Strait was probably influenced from the typhoon. Note that the day for the typhoon is in JST (UTC+9).
